# Middle-schoolers’ reading and lexical-semantic processing depth in response to digital and print media: An N400 study

**DOI:** 10.1371/journal.pone.0290807

**Published:** 2024-05-22

**Authors:** Karen Froud, Lisa Levinson, Chaille Maddox, Paul Smith

**Affiliations:** Neurocognition of Language Lab, Department of Biobehavioral Sciences, Teachers College, Columbia University, New York, New York, United States of America; University of Sydney, AUSTRALIA

## Abstract

We report the first use of ERP measures to identify text engagement differences when reading digitally or in print. Depth of semantic encoding is key for reading comprehension, and we predicted that deeper reading of expository texts would facilitate stronger associations with subsequently-presented related words, resulting in enhanced N400 responses to unrelated probe words and a graded attenuation of the N400 to related and moderately related words. In contrast, shallow reading would produce weaker associations between probe words and text passages, resulting in enhanced N400 responses to both moderately related and unrelated words, and an attenuated response to related words. Behavioral research has shown deeper semantic encoding of text from paper than from a screen. Hence, we predicted that the N400 would index deeper reading of text passages that were presented in print, and shallower reading of texts presented digitally. Middle-school students (*n* = 59) read passages in digital and print formats and high-density EEG was recorded while participants completed single-word semantic judgment tasks after each passage. Following digital text presentation, the N400 response pattern to moderately-related words indicated shallow reading, tracking with responses to words that were unrelated to the text. Following print reading, the N400 responses to moderately-related words patterned instead with responses to related words, interpreted as an index of deeper reading. These findings provide evidence of differences in brain responses to texts presented in print and digital media, including deeper semantic encoding for print than digital texts.

## Introduction

The use of digital platforms for delivery of instruction and information at school and at home is now requisite for students at all levels, from elementary school through higher education. The increased use of digital materials alongside paper-based materials in learning environments has motivated research into the efficacy of reading and learning in one format versus the other (e.g., [1–4]), and although there is an overall finding for a paper-based advantage, the outcomes have been characterized by small effect sizes and variability associated with participant, stimulus, and task factors (e.g., [5, 6]). Some reports have indicated no differences between print and digital media with respect to story understanding or comprehension measures [7–10], or test scores [11]. An eye-tracking and EEG investigation [12] revealed no differences across media in fixation times or theta oscillatory power (a correlate of memory encoding) for younger adults, although older adults showed shorter mean fixations and lower EEG theta power when reading from tablet computers compared to e-readers or paper.

As noted by Clinton [1], reports of reading times in different mediums have been inconsistent, with some authors reporting longer reading times for paper than digital environments (e.g., [13–16]), others reporting the reverse [17, 18], and yet others finding no difference between media [7, 19]. Effects of reading time on comprehension accuracy are also mixed. Some of those reporting shorter reading times for computer-based reading also reported a decrease in reading comprehension accuracy in this medium [14, 15]; although Chen and Cantrambone [13] reported shorter reading times for digital texts but no difference in comprehension between mediums; while Singer Trakhman et al. [16] found differences between distinct reading profiles with respect to both speed and comprehension, with overall scores reflecting longer processing times and lower accuracy in digital mediums. However, Kim and Kim [20] found that teenagers read faster in the paper-based condition compared to a digital format with a scrolling feature, and also that they scored significantly higher on exams when they studied via paper-based texts.

Others [1, 19] reported no difference in reading times between the two media but observed higher comprehension scores in the paper-based condition, suggesting a metacognitive moderating factor. This proposition is supported by results showing that outcomes are poorer on computer-based exams when time is constrained, in contrast to self-paced exams, perhaps because students find it more difficult to self-regulate, monitor task progress, and manage goals and time in digital space [21–23].

The effects of medium on metacognitive regulation of learning have been further investigated in a series of studies [22, 24, 25]. Lauterman and Ackerman [25] evaluated differences between mediums under distinct learning constraints (time pressure, interrupted study, and free regulation). Their sample showed no effect of medium when participants were permitted to freely regulate their learning activities, but there was a clear screen inferiority effect under time pressure. These findings interacted with participants’ stated preferences for digital vs. paper-based learning, such that those who preferred digital formats were able to achieve similar results when learning via screens and via paper. However, paper learning still conferred an advantage; efficiency of learning dropped over time for participants working from screens, whereas participants learning from paper maintained learning efficiency throughout the study process. Metacognitive effects of reading medium in adolescent readers were investigated by Ronconi et al. [26], who pointed out the importance of this age group for reading research. The pervasive use of screens and digital media by adolescent students, in and outside of school, has been exacerbated by the pandemic-related shift to long-term distance learning. In addition, motivation for reading has been observed to decrease in this age group as they transition from elementary to middle and high school (described by Chall & Jacob [27] as “the fourth grade slump”; see also [28]). In their study of adolescent reading on tablets vs. printed page, Ronconi et al. [26] measured reading time, reading comprehension (at three levels: main idea, key points, and related information), and calibration bias–that is, the difference between participants’ judgments of their own comprehension performance, and their actual performance. Although reading speed was not affected by medium overall, findings were mediated by sex of the participants, with boys reading faster from screens than paper and also showing greater calibration bias than girls during screen reading. Calibration bias was found to mediate effects of reading medium on text comprehension, at the levels of main idea and key points.

In addition to metacognitive factors, reading comprehension seems also to be moderated by the level of analysis required of participants; for example, recall of overall gist or main idea versus specific details from a written passage. Comprehension scores measured by understanding of gist have been repeatedly shown not to differ between narrative and expository texts, regardless of the medium of text presentation [1, 2, 6, 15], although Ronconi et al. observed a print advantage for comprehension at the level of main idea. The effects of text genre (expository or narrative) are also mixed; for instance, Mangen et al. [29] observed a comprehension advantage for print over digital media for both narrative and expository texts, whereas others have shown that reading narrative texts yields similar comprehension outcomes regardless of the medium [1, 2, 30]. However, in a recent meta-analysis, Salmerón et al. [4] showed that level of comprehension (textual and inferential) was not moderated by text genre (narrative or expository), whether the texts were read on a tablet/computer or paper.

These varied outcomes may be attributed to a number of factors, such as differences in age and grade-level of study participants, their learning goals, and learned strategies. For elementary students, medium of presentation has been shown to have little influence on comprehension of simple texts [7, 8]. Lenhard et al. [14] found that elementary and middle school children were faster at completing a reading comprehension assessment on computer compared to paper under time constraints, but at the expense of accuracy. Støle et al. [31] evaluated the effects of medium (computer vs. print) with 10-year-olds, and found that participants’ reading comprehension scores were higher on paper-based than computer-based tests; this held true across low, medium and high comprehension skills. Critical reading skills of high school and college students were compared by Eshet-Alkalai and Geri [32], who found that younger students performed better when reading news in digital formats compared to paper, while college students performed better on the same task when reading in paper formats.

Against this lack of clarity in the behavioral findings, there has been little brain imaging work to further elucidate the mechanisms that underpin reading in print versus digital formats. Two studies used functional near-infrared spectroscopy (fNIRS) to investigate memory encoding and retrieval processes supporting learning [33, 34]. Lee et al. [33] examined differences in neuronal efficiency during encoding and retrieval of information read from paper versus a digital tablet. Participants read and memorized a mini-novel (the information encoding phase) and then completed multiple choice questions (the memory retrieval phase). Concentrations of oxygenated hemoglobin (HbO) were measured in areas of the prefrontal cortex (PFC) associated with working memory. Performance on the multiple-choice task did not differ between mediums, but lower HbO concentrations were observed for the paper-based group during encoding, suggesting greater encoding efficiency in this medium. There were no differences in HbO during the memory retrieval phase. Anuardi et al. [34] conducted a reading span task on paper and tablets, and participants were asked to memorize an underlined word in each presented sentence. Scores on the reading span task for the two mediums were equivalent, but again prefrontal cortex activity was lower during task performance in the paper-based condition than the tablet condition, suggesting greater resource efficiency when learning from paper.

Umejima et al. [35] also investigated encoding and retrieval from reading in different media, using functional resonance imaging (fMRI). For the encoding phase, participants read appointment information in three conditions: paper notebooks, digital tablets, and mobile phones. They then entered the information on a calendar displayed in each medium. For the retrieval phase participants responded to a series of multiple-choice questions about the scheduled appointments, while fMRI data were recorded. During encoding, participants were much quicker when making calendar entries in notebook than digital formats. During retrieval, neither overall accuracy nor response times for the memory retrieval test questions differed significantly among the three groups, though accuracy for simple recall questions was significantly higher for the paper notebook group compared to the others. Brain activations during retrieval were significantly higher in areas of the pre-frontal cortex for the notebook compared to tablet and phone conditions. Umejima et al. suggest that together the outcomes indicate a possible advantage for information encoding when working in the paper notebook format.

Relevant to the current study, a few studies have applied electroencephalography (EEG) methods to explore the neurocognitive processes underlying reading in different mediums. Kretzschmar et al. [12] recorded electroencephalography (EEG) during their eye-tracking paradigm, that was designed to evaluate whether stated preferences for the printed medium (versus one of two digital devices) correlated with indices of text engagement in young and older adults. Comprehension accuracy did not differ with text presentation medium for either group, but the older adults showed shorter mean fixation durations and lower EEG theta band voltage density when reading from a tablet computer in comparison to an e-reader or a printed page. Younger adults did not show any such differences, and Kretzschmar et al. interpreted the observed differences as relating to limitations on memory encoding and retrieval for the older adults, affected by reduced contrast sensitivity, that could be somewhat ameliorated by the backlit display of the tablet computer. In another EEG study comparing brain responses to reading in different mediums, Zivan et al. [36] examined brain activation differences in EEG power bands for 6-8-year-olds reading expository texts from screens versus printed paper. They observed a higher theta-beta ratio for screen reading, an index of greater difficulty in attentional allocation to a given task. This built on earlier observations by Zivan et al. [37], who had observed increased theta-beta ratios in preschoolers who were exposed to story-telling via screens during a 6-week period compared to those who were exposed to the same stories read live. To the best of our knowledge, however, there exist currently no reports of event-related potential (ERP) measures applied to the question of reading in different media, specifically print vs. digital text processing in children.

For our investigation, we drew upon depth of processing theory, first posited by Craik and Lockhart [38]. The premise of this theoretical framework is that shallow information processing yields less durable episodic memory traces, while deeper processing results in more durable traces. The central claim is that the more deeply information is processed, the more durable the associated memory traces. Kintsch [39–41] has described text comprehension as a dynamic process of constructing meaning from semantic relations among words in the text and stored knowledge about subject matter. According to seminal work by Craik and Tulving [42], processing of verbal text information requires the use of semantic processes (protocols concerning the ways in which words work together to create meaning); hence, text processing strategies for reading may involve drawing on contextual, semantic, grammatical, and phonemic knowledge in systematic ways to work out what information is conveyed by a text. Such strategies would allow an encoded unit to be integrated with knowledge of the world or “semantic memory” (e.g., [43]). At retrieval, informational cues would then tap into this semantic memory structure to reconstruct an initial encoding [42]. Likewise, Kintsch [39] proposed that lexical-semantic information is integrated into comprehension via activation in a network of related concepts. As semantic activation spreads within such networks, it can facilitate word recognition as well as supporting connections between inferentially-related or overlapping propositions.

There exists a large amount of evidence suggesting that text processing is typically shallow, much of it based on psycholinguistic experiments demonstrating the difficulty of identifying local and global anomalies in texts (e.g., [44–47]). However, different factors may influence the relative depth or shallowness of lexical-semantic processing of words. Using text-change tasks wherein words in a passage are changed on a second reading and participants are asked to identify the altered word, Sturt et al. [48] demonstrated that depth of processing is affected by interactions between linguistic focus and semantic distance, while Sanford et al. [49] showed that syntactic complexity is also a factor affecting depth of lexical-semantic processing with failure to recognize the change indicative of shallow lexical-semantic processing for that lexical item. Based on this framework, we propose that *the medium* whereby readers engage with text/reading material could also be a crucial determinant of differences in depth of lexical-semantic processing, and consequently the durability of the semantic memory structure that is established in response to a written text. Congruous encoding between a semantic structure already established by a reader and a semantic structure associated with a newly encoded unit, as conceptualized in models like that developed by Kintsch [39], should facilitate efficient comprehension of a text, first because a meaning-referenced elaborated trace network is formed, and second because robust congruent semantic encoding also entails alignment with the structure, rules, and organization of semantic memory [42, 50].

Consistent with this view of semantic structure and encoding processes, we hypothesized that depth of lexical-semantic processing is key for reading comprehension and for congruency between existing semantic structures and the semantic structures encoded by probe words. Based on previous research, semantic encoding of text presented on paper is deeper than that of text presented digitally [1, 29]. Therefore, our experimental approach to measuring reading comprehension in the brain made use of a signature of electrophysiological activation associated with semantics in language processing: the N400 event-related potential (e.g., [51, 52]).

The N400 event-related potential (ERP) indexes brain response differences between expected and unexpected stimuli. Since we hypothesized that the encoding of word meaning during the reading experience is critical for comprehension, then we should be able to index shallow vs. deep information processing of text delivered in print or digitally by observing differences in N400 responses to probe words that were selected to be related, moderately related, or unrelated in meaning to written passages. Based on this hypothesis and given that both the culturally prevailing view and data meta-analytic studies [1, 2, 21, 53, 54] suggest that reading digitally presented text promotes shallower engagement than print, our predictions for the electrophysiological index were as follows: 1) In the digital reading condition, the N400 amplitude response to related word probes was predicted to be attenuated compared to moderately related and unrelated word probes, with amplitude differences between moderately related and unrelated word conditions expected to be equivalent; and 2) In the print reading condition, the N400 amplitude response to the three conditions is predicted to be graduated. Specifically, the amplitude measures were predicted to increase in their negativity such that the response to the related words would be most attenuated, followed by the moderately related words, with words that are unrelated to the text passage eliciting the greatest negativity. Differences in the N400 ERP response between the two mediums for the moderately related word conditions may offer essential insights about the neurocognitive processing underlying reading comprehension, and whether readers in some situations process text somewhat more shallowly under conditions of digital text presentation than when processing text via print presentation.

## Materials and methods

### Participants

We collected data from 65 participants from the New York City metropolitan area and were able to retain data from 59 (five were removed due to unusable behavioral data; one was removed due to low numbers of EEG trials per condition following artifact detection–detailed further below).

The mean age of retained participants was 10.88 years (*SD* = 0.77); of these, 28 identified as male and 28 as female, with one participant giving no response to this question. Most participants were in 5th (*n* = 21) or 6th grade (*n* = 22) at the time of their lab session, as expected; the remainder were in 4^th^ (*n* = 2), 7^th^ (*n* = 10), or 8th grade (*n* = 2), and two indicated “other”. All participants were from households with at least one parent or guardian who attended some post-secondary education, with the majority having earned degrees: associate degree (3.5%), bachelor’s or undergraduate degree (28.1%), master’s degree (52.6%), or doctorate (10.5%). Household annual income was reported as $150,000 per year or above for 56% of participants, with the balance of participants spread among the other income brackets (no response; $35,000 –$49,999; $50,000 –$74,000; $75,000 –$99,999; $100,000 –$149,999).

### Stimuli

#### Passages

Based on the key finding that a paper-based reading advantage is seen largely in studies using informational or a mix of informational and narrative text [1, 2, 15], all reading passages were developed as informational texts. Several additional goals were set for the passage development so that passages could be used as controlled experimental stimuli yet remain similar to text that might be found in a classroom setting. The passages covered a range of topics to account for differing interests among participants. We also controlled the level of reading difficulty and complexity while maintaining grade-level and age-appropriate standards. Finally, we ensured that there was sufficient content for generation of word probe stimuli for the subsequent single-word semantic relatedness judgement task. These passages were limited to relatively simple sentence structures (minimizing relative or subordinate clauses) while preserving the historical and scientific accuracy of the presented material.

Eight passages were created in thematic pairs to allow for later comparison across mediums. The passages were matched for length with respect to average number of words per sentence (*mean* = 11.736, *SD* = 1.073), number of total words (*mean* = 189.125, *SD* = 9.250), and number of sentences (*mean* = 16.250, *SD* = 1.389). Readability scores were calculated and matched for each passage, specifically the Flesch-Kincaid Grade Level [55] (*mean* = 5.775, *SD* = 0.711), Gunning Fog score [56] (*mean* = 7.950, *SD* = 0.795), and the SMOG index [57] (*mean* = 6.388, *SD* = 0.541). In addition, we matched the passages on Propositional Count (PC), a quantification of the number of semantic units and their connections within the text [58–60] (*mean* = 65.750, *SD* = 2.188).

#### Passage reading comprehension measure

To assess participant comprehension for each text, it was necessary to develop passage-specific assessments. The Sentence Verification Technique (SVT; [61]) is an assessment procedure based on the theoretical assumption that reading comprehension is a constructive process involving interactions between incoming discourse and the reader’s prior knowledge structure. SVT comprehension test items are graded questions derived from texts that require varying levels of passage knowledge to answer. The four question types specified within the framework are: *Explicit/Original*, whereby a sentence directly from the text must be identified as such by the reader; *Paraphrase*, whereby a sentence from the text is paraphrased, and must be identified as such; *Meaning Change*, a sentence that changes an aspect of meaning presented in the text, and which should therefore be rejected by the reader; and *Unrelated/Distractor* items. We used *Explicit* and *Unrelated* categories from the SVT framework as defined but made adaptations to the other two question types. For the *Meaning Change* condition, we altered sentence meanings by replacing only a single propositional predicate with a related probe word. Our *Paraphrase* items were not sentences from the passage themselves, but true statements that combined propositions from across the entire text. SVT sentences were constructed to minimize syntactic complexity (active sentences only, no subordinate clauses), matched for sentence length (mean 10.625 words per sentence, *SD* = 1.619), and controlled with respect to the age of acquisition (AoA) of individual words (based on ratings from [62]; mean AoA for all SVT items = 5.984, *SD* = 1.936).

Conventionally, SVT items elicit a binary response (*Yes*, *No*), making scoring a simple process. For our purposes, we provided students with three selection options based on the relatedness of the sentence to the passage: (1) *I read exactly this sentence in the passage*; (2) *The facts in this sentence were in the passage*; or (3) *None of the facts in this sentence were in the passage*. We applied a binary scoring procedure to *Explicit* and *Unrelated* responses to SVT items: an *Explicit* item was scored correct if response (1) was selected, and an *Unrelated* item was scored correct if response (3) was selected. In the conventional SVT framework, *Meaning Change* test items should all be identified as false, whereas our items were a mixture of true and false statements. Per convention all *Paraphrase* items were true. For analyses, we marked a *Paraphrase* item as correct if a respondent indicated response (2); we scored the *Meaning Change* items as correct if either (2) or (3) was chosen, depending on the assigned truth value for that statement.

#### Validation of passages and passage comprehension items

Prior to conducting the experiment, we collected online reader response data to the eight passages via Panelbase LLC (panelbase.net). These data ensured that stimuli were balanced with respect to the following parameters: reading time for each passage; participant interest in the passages; self-reports of reading difficulty; a set of cloze questions to evaluate attention to each passage; and the constructed SVT items. Respondents represented a random sample of students matching the study target population, drawn from U.S. urban areas excluding New York City. Between 70 and 80 participants completed a survey that included a selection of two of the eight passages. The results of this pre-study validation procedure pointed to general equivalency across these eight passages in terms of difficulty and accessibility, as well as general responses to the SVT question types. Analyses of this data identified that one passage set (two thematically related passages) was more difficult relative to the others, and so these two passages were excluded from the experiment.

#### Stimulus probe words

Probe words for the semantic relatedness judgment paradigm were generated by identifying verbs or nouns at the center of propositions in each passage as targets for semantic field interrogation. Using the WordNet 3.0 database [63–65] each selected verb and noun was used as a search term and the relevant propositional sense was identified in the returned synset listings. Each synset was then expanded and lexical items (uninflected, nonderived) of the same word class as the target proposition were selected from synset lists. Frequency (Zipf scores: [66]), age of acquisition (AOA: [62]) and length characteristics (NLET, NPHON, and NSYLL, all from the MRC Psycholinguistics Database: [67]) were determined for each item. Items were included in the semantic rating experiment only when their frequency and AOA ratings were within 1 SD of the mean for the target passage. Concreteness was also evaluated, and there were no differences between any of the word conditions with respect to that property [68]. Probe words fell into three categories: *related*, *moderately related*, and *unrelated*.

Potential items for the *related* category of word probes were identified based on the specific propositions identified in each passage. Based on the number of related words that were identified per passage, a number of words from a pool of semantically *unrelated* words that were likewise matched on AOA and frequency were also included, to yield up to 100 target items per passage. Relatedness ratings were validated using the online platform Prolific (prolific.co). Semantic ratings were solicited from an adult population as opposed to the target population of middle-school students given that adults are more likely to have well-developed semantic networks [69]. Adult raters read each text passage and then rated candidate probe words for relatedness to the passage on a scale from 0% to 100%. Each participant rated potential probe words for two passages. For each passage, the candidate probe words were rated on relatedness by 100–150 participants. Ratings were trimmed to remove ratings of 0% or 100% and Gaussian mixture modeling (e.g., [70, 71]) was applied to data for each passage to cluster ratings into the three stimulus categories (related, moderately related and unrelated). The 20 words closest to the mean score within a cluster were assigned to that category; if a category contained fewer than 20 words, only that many words were assigned. Probe words that applied to multiple passages were assigned to the category and passage for which they were closest to their cluster mean, and the next closest word was chosen for the other passage.

The *moderately-related* words, those that fell within the center cluster, were labeled as “chimera” items, reflecting the possibility that a word that is moderately related to some context could also be identified as moderately unrelated to that context. The judgement task for N400 elicitation required a binary decision concerning relatedness (related vs. unrelated), and these items were evaluated as somewhere in between. The chimera words were crucial to our predictions with respect to the depth of lexical-semantic processing, as we anticipated that deeper processing would facilitate the identification of chimera words as being related to the preceding textual context, while shallower processing would be more likely to result in identification of chimera words as unrelated.

#### Psychometric measures

All participants completed a set of standardized assessments, plus an additional assessment of auditory working memory, as follows:

- Wechsler Intelligence Scale for Children V [72] Digit Span subtest–an assessment of working memory capacity
- Woodcock Reading Mastery III [73] Passage Comprehension subtest–an assessment of general reading comprehension
- Woodcock Reading Mastery III [73] Word Attack subtest–an assessment of phonemic decoding ability
- Swanson Listening Sentence Span Task (LSST; [74])–an assessment of working memory span that is mediated by language

### Data collection

Data were collected in three phases: Phase 1 for the online administration of psychometric assessments; Phase 2 for the EEG recordings and the immediate passage recall comprehension measure; and Phase 3 for the online administration of the passage retention comprehension measure. All informed consent and experimental procedures were carried out with approval of the Teachers College, Columbia University Institutional Review Board (Protocol # 22–173). Study recruitment began in February 2022 and continued until March 2023. Written informed consent for participation in the study was obtained from the parent or guardian of each minor participant, and both verbal and written assent were obtained from all individual participants directly. There were separate approved informed consent and assent forms for each of the three study phases.

#### Phase 1: Psychometric assessments

In Phase 1, responses to behavioral assessments were collected by two trained assessment administrators online during video conference sessions. The parent/guardian received a study overview, consent, and assent forms in advance of the scheduled appointment. They were asked to select a quiet setting with the home with minimal distractions where the participant could complete the assessments. Online, the assessment administrator reviewed the materials and responded to questions before the parent/guardian and their child completed the consent and assent forms via a Qualtrics survey. The sessions were approximately 25 to 30 minutes in length and audio recordings were stored for the purpose of second-scoring of measures.

#### Phase 2: EEG recording

In Phase 2, participants and their accompanying parent/guardian attended the Neurocognition of Language Lab at Teachers College, Columbia University. Informed consent and assent procedures were conducted, this time via face-to-face conversations (for the verbal assent of minors) and paper-and-pen administration (for written consent from parents/guardians and written assent from minors). High-density EEG data were continuously recorded in NetStation 4.3.1, using a 128-channel HydroCel Geodesic Sensor Net (MagStim Electrical Geodesics, Inc.). Signals were amplified using a NetAmps 200 series amplifier. Samples were collected at a rate of 500 Hz; an online low-pass filter of 200 Hz and high-pass filter of .1 Hz were applied. Impedances were kept below 40 kilo-ohms and were re-checked between blocks. Participants completed sessions in an electrically shielded and sound-attenuated room, seated 65 cm from a computer monitor with a brightness of 75 cd/*m*^2^.

Each participant was first exposed to texts that were presented via either a paper booklet (print) or a laptop screen (digital). For the digital reading condition, visual readability variables (contrast, brightness, text size) between the laptop screen and the stimulus presentation screen were held equivalent. The order of medium and passage presentation was balanced between participants. We counterbalanced the order of medium (digital-first vs. print-first), and the sequence of presentation for the themed pairs of passages. With this approach, we were able to systematically vary the order of passage presentation across participants, ensuring that each individual passage appeared an equal number of times in each medium, and that each themed pair appeared equally often as the first, second, or third set of texts read by a participant. For each passage, reading time was recorded, and then participants completed two tasks, presented using E-Prime 3.0 (Psychology Software Tools, LLC).

**Fig 1:**
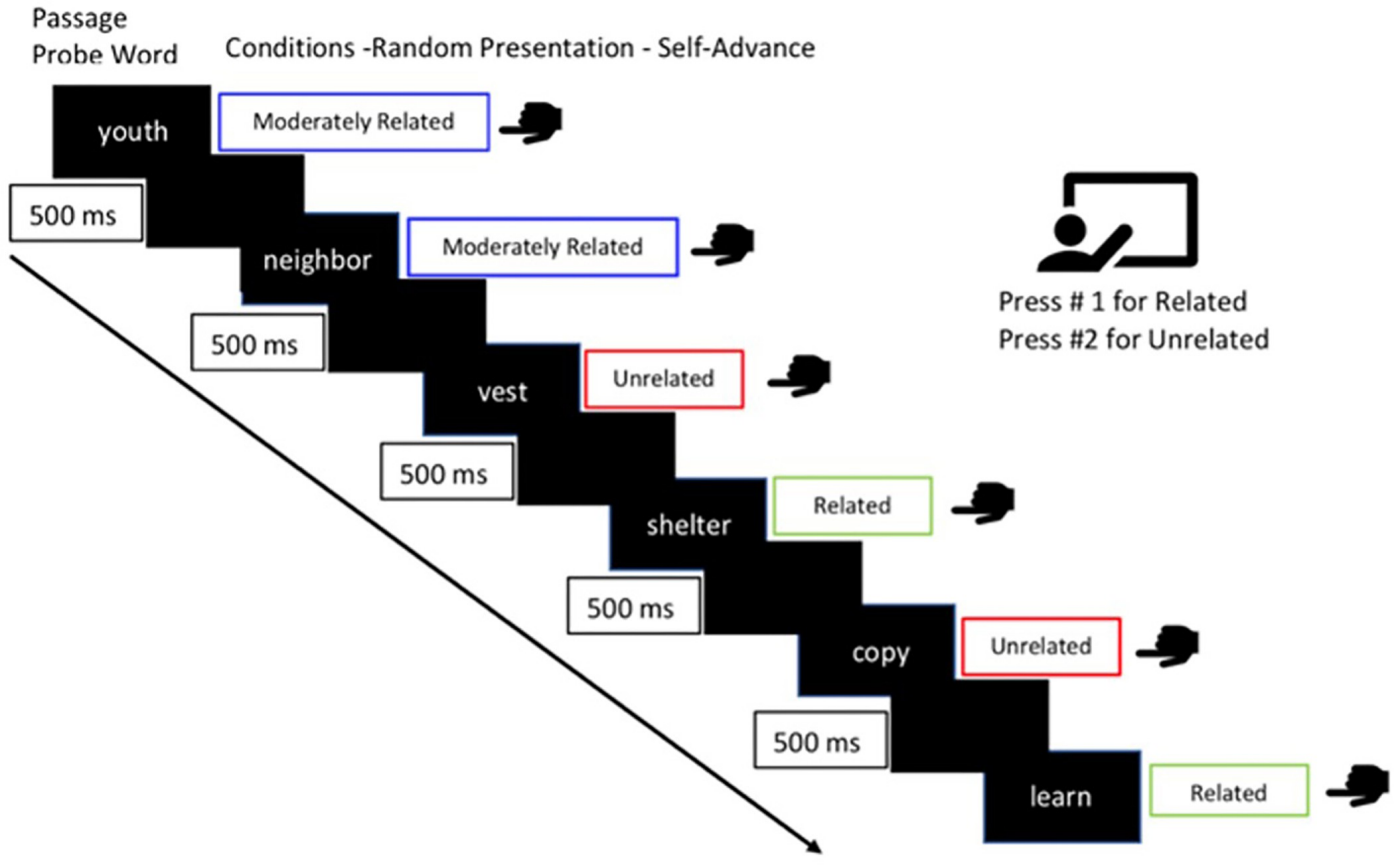
Timeline for example trials from the semantic relatedness task.

First, participants read one text passage in their assigned first medium. Then they completed the semantic relatedness judgment task in response to single probe words presented on a computer screen. They responded to each word by pressing one button to indicate that a word was related to the passage, and another if they thought the word was unrelated (see Fig 1).

After the semantic relatedness judgment task, the SVT recall comprehension test items were displayed, and participants were prompted to respond. This procedure was repeated for two additional passages in the selected medium (either print or digital). Then, the medium of presentation was switched, and the process was repeated for another three passages.

#### Phase 3: Passage retention measure

Following the EEG recording session, participants were emailed a link to the follow-up Qualtrics retention comprehension survey. As in Phase 1, consent and assent were indicated via Qualtrics survey questions prior to participants completing the comprehension items online. This assessment consisted of the same SVT recall comprehension test items that they had completed during the EEG portion of the study, and was included to provide an indication of information retention. Participants were asked to complete the measure within 24 hours of their visit to the lab, but survey responses were accepted up to seven days after their lab visit.

### EEG data analysis

#### Pre-Processing

EEG data were pre-processed using the Harvard Automated Processing Pipeline for Electroencephalography (HAPPE; [75]), specifically the event-related extension (HAPPE+ER; [76]). The sensitivity of the HAPPE procedures allows for more trials to be kept and averaged when dealing with high-variance data such as those associated with children. Globally bad channels were detected and removed from the remainder of the pipeline. Across all participants, an average of 93.6% (SD: 4.4%) of channels were good, with a range of 61.2% to 99.2%. A hard wavelet threshold was applied to remove artifacts from the continuous EEG data, a technique that improves upon previous methods of detecting artifacts to retain more of the EEG signal instead of rejecting segments at this stage [76]. A pre-established bandpass filter from 0.1–40 Hz was utilized, and data were segmented from 100 milliseconds (ms) before stimulus presentation to 750 ms post-presentation.

Segmented data were subjected to baseline correction, whereby the average of the EEG recorded during the baseline period for each epoch was subtracted from the post-stimulus period. Bad data within each segment were interpolated and segments were rejected based on a joint probability criterion as well as amplitude cutoffs of -150 and 150 microvolts. Globally bad channels were replaced based on spherical spline interpolation of data from surrounding electrodes, and data were re-referenced offline to the average of the left and right mastoid channels (electrodes 57 and 100).

Participants were excluded from further analysis if more than 40% of trials for any passage were rejected. Of 65 participants, one was excluded due to low numbers of trials in the final analysis and others due to inability to use behavioral data; analyses were therefore based on data from 59 participants. For all retained participants, at least 50% of trials were deemed usable; on average, 66.5% of trials were usable (SD: 5.8%; range: 53.2% to 82.3%). The numbers of trials per participant did not vary significantly across medium or passage. For the related and unrelated conditions, error trials (trials in which a participant had misidentified a related word as unrelated, or vice versa) were also excluded from further analysis. All trials were kept for the chimera condition, as their intermediate level of relatedness makes them hard to accurately categorize in a binary fashion. In the print medium, 910 related trials, 2,024 chimera trials, and 1,961 unrelated trials were used in subsequent analyses; in the digital medium, trial numbers came to 909 related trials, 2,016 chimera trials, and 2,028 unrelated trials.

Baseline-corrected epochs for each word condition were then averaged together for each individual participant, providing individual averages per medium and condition. Individual event-related potentials were interrogated for mean amplitude of the target component within an *a priori-*established time window, 300–500 milliseconds post-stimulus. Individual averages per condition were then grand averaged to generate group ERP waveforms.

#### Montaging

**Fig 2:**
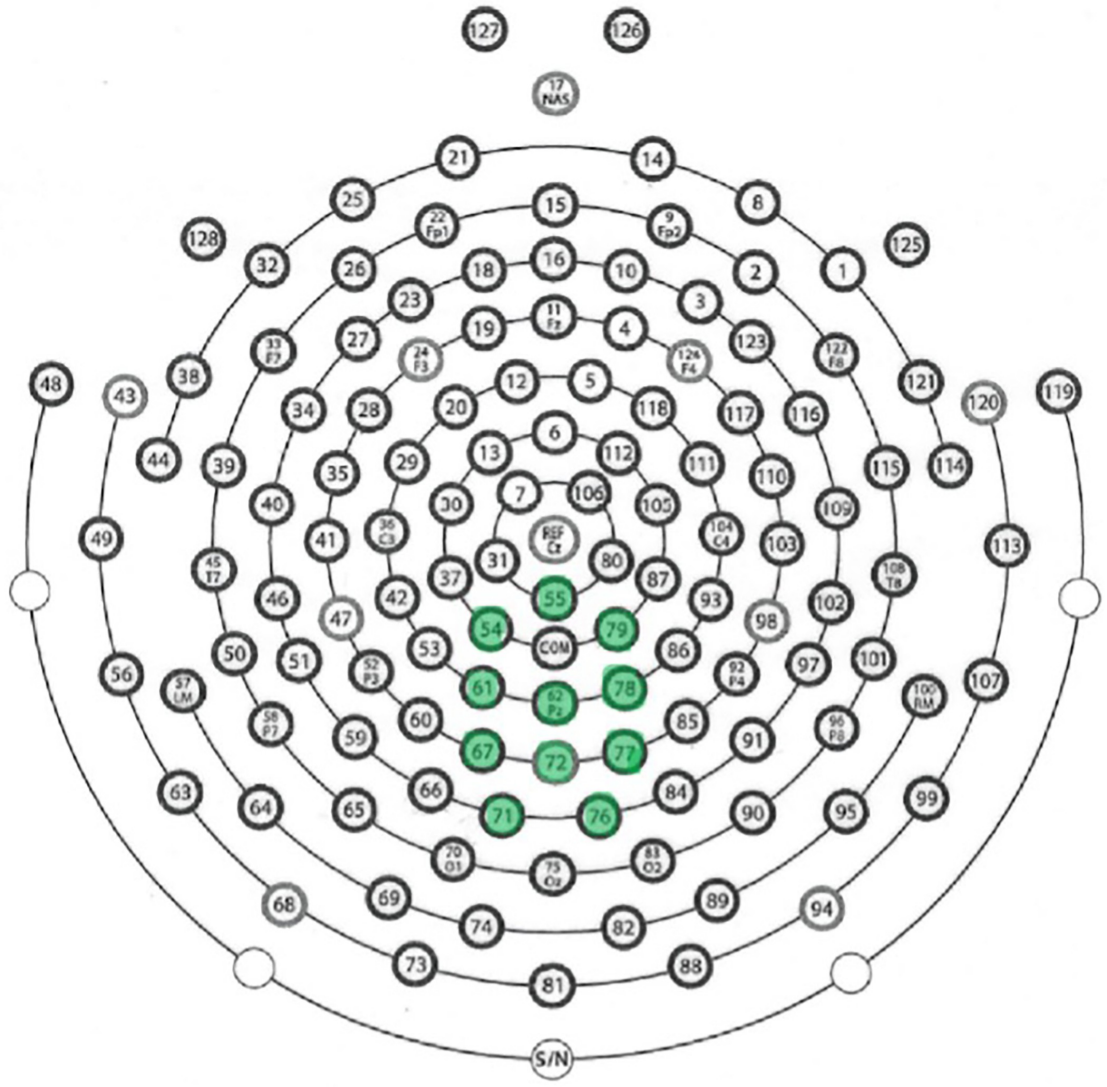
Montage for N400 analysis. Electrodes included in the analysis montage are indicated in green: electrode numbers 54, 55, 61, 62, 67, 71, 72, 76, 77, 78, 79.

N400 montages vary across studies (e.g., [77]). The electrode montage for investigation of the N400 component was selected based in part on the N400 context and discourse literature [78–84]. Fig 2 below indicates the montage of interest; all plots of the derived event-related potentials relate to this montage.

## Results

### Phase 1: Psychometric assessments

All participants completed a set of standardized assessments, plus an assessment of auditory working memory. Table 1 below provides mean scores and standard deviations for each assessment for all included participants (*n* = 59). We applied a criterion to include only those participants whose scores on all assessments were within 3 standard deviations of the sample mean for each assessment. All participants met this criterion. While we did not have any outliers that needed to be removed from the data analysis, a range of abilities was represented within this sample of middle school students.

**Table 1:** Mean scores and standard deviations for psychometric reading assessments.

*Source Assessment Battery*	*Subtest / Scoring Sample*	*Mean standard score (SD)*
*WISC-V*	Digit Span–forwards	11.49 (3.28)
	Digit Span–backwards	10.93 (3.37)
*WRMT-III*	Word Attack–by grade	107.20 (11.89)
	Word Attack–by age	107.90 (11.70)
	Passage Comprehension–by grade	116.05 (14.44)
	Passage Comprehension–by age	117.81 (14.76)
*LSST*		2.10 (1.34)

WISC-V, Wechsler Intelligence Scale for Children, 5^th^ edition. WRMT-III, Woodcock Johnson Reading Mastery Test, 3^rd^ edition. LSST, Swanson Listening Sentence Span Task.

We evaluated correlations between these measures to determine which assessments of working memory (digit span and LSST) were correlated with the measures of language skill from the WRMT-III. A review of the relationships between different measures revealed no significant correlations between the Listening Sentence Span Task (LSST) and traditional working memory measures (forward and backward digit span: *r* = .088, *p* = .507 and *r* = .155, *p* = .241, respectively). However, there was a significant correlation between the digit span scores (*r* = .559, *p* < .001). The LSST measure was found to be positively correlated with both word attack and passage comprehension scores (see Table 2, below).

**Table 2:** Correlations between scores on working memory and language assessments.

*WRMT-III subtest*	*LSST* r *(*p*)*	*Digits Forward* r *(*p*)*	*Digits Backward* r *(*p*)*
*Word Attack–Grade*	.354 (.006)**	.510 (< .001)**	.408 (.001)**
*Word Attack–Age*	.327 (.011)*	.517 (< .001)**	.418 (< .001)**
*Passage Comprehension–Grade*	.327 (.011)*	.261 (.046)*	.401 (.002)**
*Passage Comprehension–Age*	.326 (.012)*	.241 (.065)	.399 (.002)**

WRMT-III = Woodcock Johnson Reading Mastery Test, 3^rd^ edition; LSST = Swanson Listening Sentence Span Task. Correlation coefficients (*r*-statistics) are provided with *p*-values.

* = significant at < .05

** = significant at < .01.

These findings indicate that (a) working memory, as measured by both the Listening Sentence Span Task (LSST) and the digit span Forward and Backward subtests, was within a typical range across the group of participants; (b) that working memory is important to control in experimental approaches to reading comprehension; and (c) that working memory is *not* likely to be a factor influencing neurophysiological response differences between passages in this experiment.

### Phase 2: Event-Related potentials

**Fig 3:**
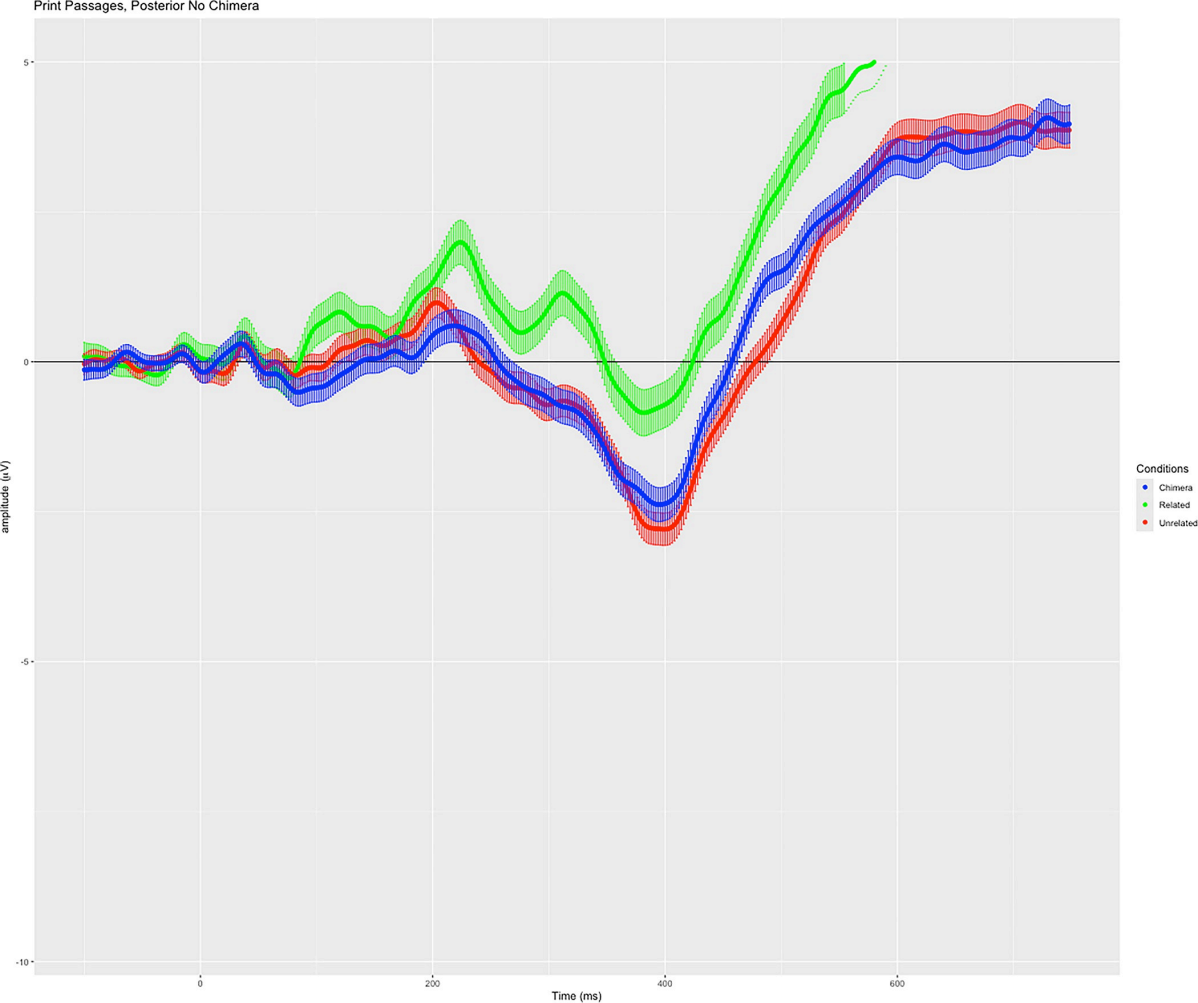
Grand-averaged waveforms in response to the semantic relatedness task following digital text presentation. Includes all retained participants, correct response trials only for related and unrelated word conditions, and all responses to chimera words (no error criterion for this condition). Variance around the mean waveforms is shown as shadow. Green: Related condition; blue: Chimera condition; red: unrelated condition.

We examined the grand-averaged N400 responses to all probe word conditions (i.e., related, chimera, unrelated) within each medium (i.e., following texts presented in digital vs. print media). Plots displaying the grand-averaged waveforms of participant responses for each probe word condition within each presentation medium condition are shown in Fig 3.

A mixed model two-way ANOVA was conducted to compare the factors of text medium and probe word category. No significant main effect of medium was detected (*F* (1, 58) = .158, *p* = .692, *η*^*2*^ = .003), but the main effect of word category was significant (*F* (2, 116) = 13.533, Greenhouse-Geisser corrected *p* < .001, *η*^*2*^ = .189). Across both mediums, N400 amplitudes in response to related words were significantly more positive than responses to either chimera words (mean difference = .974 μV, *t* (117) = 2.96, *p* = .011, *d* = .273) or unrelated words (mean difference = 1.624 μV, t (117) = 4.40, *p* < .001, d = .405). Amplitude values in response to chimera words were also significantly more positive than to unrelated words (mean difference = .650 μV, t (117) = 2.84, *p* = .016, *d* = .262). A significant interaction between medium of presentation and word category was also found (*F* (2, 116) = 4.017, Greenhouse-Geisser corrected *p* = .025, *η*^*2*^ = .065). Further examination of simple main effects revealed no significant differences between responses to each probe word category following presentation of either print or digital texts (*p* > .10 in all conditions).

Significant differences were observed when comparing amplitude values between word probe categories within each of the two mediums. Following text presented in the digital medium, the N400 response to related words was significantly different from the response to both chimera words (mean difference = 1.644 μV, *t* (58) = 3.562, *p* = .002, *d* = .463) and unrelated words (mean difference = 2.204 μV, *t* (58) = 4.055, *p* < .001, *d* = .527). The response to chimera words was not significantly different than the response to unrelated words in the digital text condition (mean difference = .560 μV, *t* (58) = 1.718, *p* = .273, *d* = .223). Following texts presented in the print medium, the response difference between related and unrelated words was significant (mean difference = 1.043 μV, *t* (58) = 2.755, *p* = .023, *d* = .358). The difference between related and chimera words was not significant (mean difference = .304 μV, *t* (58) = .798, *p* = 1.00, *d* = .103), but a significant difference between chimera words and unrelated words was observed (mean difference = .739 μV, *t* (58) = 2.546, *p* = .040, *d* = .332). All *t-*tests were controlled for multiple comparisons via Bonferroni correction within medium.

### Experimental results: Behavioral findings

Following each passage, participants were asked to decide whether each word shown on screen was related or unrelated to the passage they had just read. Related words were both scored as “correctly identified” if the participants indicated they were related to the passage; unrelated words were similarly coded if they were marked as unrelated. Responses are summarized in Table 3 below.

**Table 3:** Percentages of words in each probe category identified as related to the preceding text.

*Probe Word* *Category*	*Digital Text Presentation* *% Identified Related (SD)*	*Print Text Presentation* *% Identified Related (SD)*
*Related*	45.29% (21.12%)	44.18% (21.56%)
*Chimera*	15.33% (16.22%)	15.44% (14.22%)
*Unrelated*	3.08% (4.913%)	3.32% (7.561%)

A two-way repeated measures ANOVA revealed no interaction between medium and category (*F* (1, 58) = .179, *p* = .674) or main effect of medium (*F* (1, 58) = .505, *p* = .480); however, there was a main effect of condition (*F* (1, 58) = 277.261, *p* < .001). Planned comparisons (*t*-tests) confirmed significant differences in accuracy between conditions, with unrelated words being identified significantly more accurately than related words (following text reading in the digital medium: *t* (58) = -16.314, *p* < .001; print medium: *t* (58) = -15.382, *p* < .001).

Reaction times for each word were also recorded for each participant, and are summarized by probe word category in Table 4 below.

**Table 4:** Mean reaction times for each probe word category.

*Probe Word* *Category*	*Digital Text Presentation* *Mean Reaction Time [ms] (SD)*	*Print Text Presentation* *Mean Reaction Time [ms] (SD)*
*Related*	1547.06 (490.12)	1502.55 (506.42)
*Chimera*	1454.83 (472.17)	1530.48 (559.82)
*Unrelated*	1352.92 (481.73)	1319.92 (420.52)

A two-way repeated measures ANOVA revealed a significant interaction between medium and category (*F* (2, 116) = 4.278, *p* = .016). There was no significant effect of medium, confirming that reaction times to individual words following reading in print or on a screen did not differ. A significant simple main effect was found for word category (*F* (2, 116) = 23.334, *p* < .001), and planned comparisons (paired-samples t-tests) confirmed that, in the digital medium, reaction times to the related words were significantly longer than to either the chimera (*t* (58) = 3.352, *p* < .001) or the unrelated words (*t* (58) = 4.922, *p* < .001); however, reaction times did not differ significantly between chimera and unrelated words (*t* = 2.684, *p* = .005). In the print medium, reaction times to the related and chimera words were both significantly longer than to the unrelated words (related vs. unrelated: *t* (58) = 4.389, *p* < .001; chimera vs. unrelated: *t* (58) = 5.216, *p* < .001), but the reaction times were not different between related and chimera words (*t* (58) = -0.790, *p* = .433). To verify these findings, we conducted additional generalized linear mixed model (GLMM) analyses; the additional analyses confirmed that participant and item variance did not reduce the effects of any fixed factors. The GLMM is further detailed in Supplementary Materials (S1 File).

### Comprehension accuracy

#### Immediate recall comprehension task

The reading of each passage was followed by a set of eight sentence verification items to evaluate participants’ comprehension of the preceding passage. The eight items were of four different types, as described above: explicit, paraphrase, meaning change, and unrelated. These four types of questions were designed to probe different aspects of understanding of the text and different levels of difficulty with respect to recall as well as recognition of ideas and concepts from the texts.

Responses to the sentence verification items were not recorded for 9 of the 59 participants due to software malfunction during data collection. Thus, the results below include data for 50 participants. Accuracy for these items is presented below in Table 5, separated by medium.

**Table 5:** Mean percent correct responses for each sentence verification item type, immediate presentation.

*Sentence Verification* *Item Type*	*Digital Text Presentation* *% Correct (SD)*	*Print Text Presentation* *% Correct (SD)*
*Explicit*	64.33% (21.96%)	65.33% (20.16%)
*Paraphrase*	52.33% (27.56%)	54.00% (22.22%)
*Meaning Change*	30.00% (14.68%)	27.67% (18.63%)
*Unrelated*	78.33% (22.14%)	84.00% (19.33%)
*TOTAL*	56.25% (28.17%)	57.75% (28.59%)

These items were presented immediately following the EEG experimental task. Accuracy is separated based on medium of passage presentation.

A two-way repeated measures ANOVA was run to determine the statistical significance of the interaction between medium of presentation and accuracy across question types. No significant interaction was found (medium x item type: *F* (3, 147) = 0.89, *p* = .449), and the main effect of medium was also non-significant (*F* (1, 49) = 0.561, *p* = .457). However, the main effect of question type was significant (*F* (3, 147) = 85.105, *p* < .001), and planned comparisons (*t*-tests) revealed that accuracy for each of the question types was significantly different, in the following order from most to least accurate: Unrelated > Explicit (*t* (198) = 5.528, *p* < .001); > Paraphrase (*t* (192.19) = 3.586, *p* < .001); > Meaning Change (*t* (173.14) = 8.107, *p* < .001).

#### Delayed (retention) comprehension task

In addition to collecting responses to the sentence verification items about each passage immediately following presentation, we asked participants to answer the same questions again within 24 hours after completing the lab session. However, the survey responses were accepted up to 168 hours (seven days) following the lab session. The goal was to gauge retention of the information presented in the passages, and to compare retention between media. Mean accuracy for each item type is presented below in Table 6.

**Table 6:** Mean percent correct responses for each sentence verification item type, delayed presentation.

*Sentence Verification* *Item Type*	*Digital Text Presentation* *% Correct (SD)*	*Print Text Presentation* *% Correct (SD)*
*Explicit*	63.33% (22.59%)	63.33% (19.22%)
*Paraphrase*	49.33% (20.75%)	48.67% (23.77%)
*Meaning Change*	24.67% (15.87%)	25.33% (19.70%)
*Unrelated*	63.33% (29.16%)	65.67% (28.45%)
*TOTAL*	50.17% (27.45%)	50.75% (28.12%)

These items were presented 1–7 days following the EEG experimental task. Accuracy is separated based on medium of passage presentation.

The pattern of responses to the delayed sentence verification task is similar to that of the immediate recall comprehension evaluation: meaning change items were responded to with the lowest accuracy, followed by paraphrase items. In this case, the accuracy for explicit and unrelated items appears equivalent, while overall accuracy is slightly lower for delayed vs. immediate evaluation. These results were confirmed with statistical analysis. A two-way repeated measures ANOVA was conducted, and no significant interaction between medium and item type was found (*F* (3, 147) = .195, *p* = .90). The main effect of medium was also non-significant (*p* = .75), but the main effect of item type was found to be significant (*F* (2.37, 115.91) = 41.240, *p* < .001). Planned comparisons (*t*-tests) showed that accuracy for unrelated and explicit items was not significantly different, but both were responded to significantly more accurately than paraphrase and meaning change items. Accuracy of responses to the paraphrase question type was greater than to the meaning change question type.

Additionally, we sought to identify significant differences between immediate recall comprehension (SVT items presented during the experiment run) and later retention accuracy (SVT items completed via online survey after at least 24 hours elapsed). Two separate two-way repeated measures ANOVAs were run, to observe the effects of time (immediate vs. delayed) and item type separately across the two mediums. For the digital passages, a significant interaction between time and item type was found (*F* (3, 132) = 3.204, *p* = .030), and the main effects of time (*F* (1, 44) = 13.02, *p* < .001) and item type (*F* (3, 132) = 49.697, *p* < .001) were also significant. Similarly, for the print passages, there was a significant interaction between time and item type (*F* (3, 132) = 5.448, *p* = .001) as well as significant main effects (time: *F* (1, 44) = 13.020, *p* < .001; item type: *F* (2.26, 99.64) = 60.197, *p* < .001). The effects of time reflected that total accuracy was significantly higher in the immediate responses to comprehension items than for the delayed responses (*t* (397.73) = 2.187, *p* = .03), while the interaction was driven by a difference in accuracy rates for the unrelated SVT items: on average 16.67% higher when responded to immediately after the passage reading task, compared to delayed responses (*t* (180.88) = 4.698, *p* < .001). The significant main effects of item type reflected that accuracy rates continued to follow the general pattern previously observed (Unrelated > Explicit: *t* (379) = 3.669, *p* < .001; > Paraphrase: *t* (392.61) = 5.814, *p* < .001; > Meaning Change: *t* (365.09) = 11.66, *p* < .001). With respect to delayed responses, the meaning change item type again yielded significantly fewer accurate responses than all other question types (Unrelated: *t* (165.42) = 11.699, *p* < .001; Explicit: *t* (192.3) = 13.85, *p* < .001; Paraphrase: *t* (189.09) = 8.433, *p* < .001). Paraphrase item types yielded significantly fewer accurate responses than the explicit and unrelated items (*t* (197.57) = -4.671, *p* < .001; *t* (186.27) = -4.273, *p* < .001, respectively). However, responses to the explicit and unrelated items did not differ with respect to accuracy (*t* (182.24) = 0.327, *p* = .744).

To determine whether performance on the SVT was affected by working memory, we calculated correlations between performance on the each of the working memory assessments (Digit Span Forward, Digit Span Backward, and the LSST) and accuracy on the SVT items, both immediate and delayed. Spearman correlations (Bonferroni-corrected for multiple comparisons) were used due to the ordinal nature of the LSST and possible non-normal distributions of the variables. All correlations can be viewed in the Supplementary Materials to this paper (S2 File). The only correlations that remained significant when applying the Bonferroni correction were between LSST scores and immediate SVT scores, both total (*r* = .459, *p* = .000801, corrected *p* = .036) and responses to the paraphrase items (*r* = .467, *p* = .0006287, corrected *p* = .028). No other correlations between comprehension measures and working memory measures were significant. Such a result could indicate that an increased ability to recall sentences may be helpful in accurately assessing the paraphrase items, but it does not suggest that working memory was a significant mediator for the different neurophysiological responses to digital and print items in this experiment.

## Discussion

As alluded to above, this study took place against a complex background of research and environmental factors that contribute to the importance of the findings. The COVID pandemic was a time of unprecedented disruption to our educational systems, with as-yet little understood consequences for students. Amid pre-existing doubts about the impact of digital media on the development of reading and related skills, children were abruptly forced into online instruction and even more of their engagement with text, at all levels, now happens through various digital devices. These disruptions highlighted a challenge already being faced by educators: to understand how reading comprehension and learning are changing in the age of digital information. This investigation of the neural correlates of depth of lexical-semantic processing following text reading across mediums in middle-school students is the first to apply event-related methodologies to this question, and is novel in its use of the N400 as an index. We drew upon the depth of processing theory introduced by Craik and Lockhart [38] to provide a theoretical framework for the investigation, alongside Kintsch’s [39–41] view that text comprehension is a dynamic process of constructing meaning from semantic relations among words in the text and one’s stored knowledge about subject matter. We proposed that how readers engage with text/reading material may be a crucial determinant of differences in depth of processing for lexical-semantic information contained in a text, consequently affecting the robustness of semantic memory structures that are established in support of reading comprehension. We extended the standard applications of the N400 to provide an index of processing depth associated with two mediums of text presentation: digital (via a laptop screen) and print (via a printed page).

We predicted that N400 responses to single words following texts presented in digital and print formats would differ. These predictions were largely supported by the data presented above. The waveforms indicate distinct brain responses to the semantic probe words that were presented following text reading across the two mediums. Consistent with our predictions, when passages were read on a laptop (digital), responses to subsequently presented words in the chimera (moderately related/moderately unrelated) category evoked activations similar to those associated with words that were unrelated to the text. This finding can be observed in the waveforms (Fig 3), and is supported by the lack of statistical significance in amplitude differences between chimera and unrelated word responses in the digital condition. The N400 waveforms in these two conditions can be observed to differ significantly from the response to related words.

**Fig 4:**
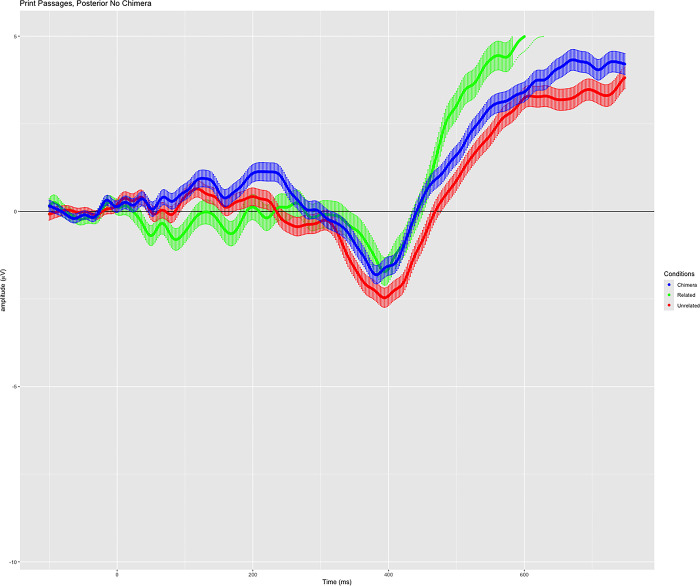
Grand-averaged waveforms in response to the semantic relatedness task following digital text presentation. Includes all retained participants, correct response trials only for related and unrelated word conditions, and all responses to chimera words (no error criterion for this condition). Variance around the mean waveforms is shown as shadow. Green: Related condition; blue: Chimera condition; red: unrelated condition.

In the print medium, we predicted that the N400 responses for the three conditions would be graduated, with unrelated words producing the greatest negativity, the response to related words being the most attenuated, and responses to chimera words falling between. However, the N400 waveform patterned differently than expected (Fig 4). Mean amplitude values within the N400 time window were significantly different between related and unrelated words, and between chimera and unrelated words–consistent with our predictions. However, contrary to prediction, the amplitude differences in response to related and chimera stimuli were not significant.

Within the context of the depth of processing theory [38–41], the primary experimental manipulation in this study related to the chimera word stimuli. Prior behavioral studies have suggested that reading on a digital device promotes shallow reading. When classifying the chimera words, we stated that these stimuli could be perceived as related or unrelated given the center clustering of their word relatedness ratings. Whether chimeras are perceived as related or unrelated to the text may depend on the strength of the encoded memory traces, and/or the lexical-semantic activations, that are established during text discourse processing. Therefore, perception of chimera words as unrelated words would be consistent with shallow lexical-semantic processing as hypothesized in digital text reading, whereas chimera words perceived as related would be consistent with deeper lexical-semantic processing as observed in print reading.

Behaviorally, there was no distinction between classification of the chimera words by study participants following the digital or print presentations of texts; in both conditions, chimera words were most frequently identified as being “unrelated” to the text. The longer reaction times to related words than words in other probe conditions likely reflect response competition (e.g., [85]), and the patterning of reaction times between chimera and related words in the print condition, and between chimera and unrelated words in the digital condition, is an expected finding given the study prediction that more robust semantic networks were expected to develop following exposure to print vs. digital texts. However, observations of the ERP responses to chimera words provide a deeper insight.

The semantic judgement task prompted participants to decide whether presented probe words were related or unrelated, potentially shaping brain responses specific to the task at hand. Therefore, how deeply a participant read the text would likely contribute to whether they perceived the chimera word probes as either related or unrelated. Keeping in mind the general expectation of shallowness in text processing [44–47], we were looking for *relative* differences in depth of lexical-semantic processing related to the medium of preceding text presentation. These seem to be borne out in the waveforms and statistics: responses to the chimera words track with responses to the unrelated words in the digital condition, and with responses to the related words in the print condition. The observed responses to the chimera word condition may index the robustness of context models for the text: if robust models are created, chimeras can be situated within the model affording greater processing efficiency, whereas when such words are situated within a less robust contextual model, as would be generated under shallower reading, the opposite would be expected. Under this interpretation, these ERP responses align with the study hypothesis and may indicate that a more “robust” semantic network was derived in response to texts presented in the print medium. Hence, we propose that the N400 brain responses observed are consistent with a finding of deeper text processing in print compared to digital media.

The increased use of digital materials alongside paper-based materials in learning environments has motivated many studies on the efficacy of reading and learning in one format versus the other (e.g., [1, 2, 6]). Investigations of reading comprehension and learning measured in terms of reading ability, reading rate, eye movement, and factual recall, have found no differences in student performance between working in the two mediums (e.g., [9, 12, 86, 87]. The present study is the first to evaluate depth of processing for print and digital informational texts in middle-school children using a brain measure (N400 ERP). Our findings contribute to this landscape by providing insights about the neurocognitive processing underlying reading comprehension. The study outcomes reveal differences in how the brain processes expository text when presented in digital and print mediums, with the former suggesting more shallow engagement and the latter conferring deeper engagement. This effect could indicate a “print advantage” with respect to depth of processing, in support of previous behavioral research [2].

### Study limitations and delimitations

As with any study that seeks to break new ground, there are important limitations to acknowledge and address in future work. Our study sample, despite our recruitment efforts, was skewed towards higher parental income and higher parental education levels and therefore does not adequately represent the diversity of the target populations (NYC metropolitan area). Future work should direct efforts towards recruitment of participants from a wider range of SES and parental educational backgrounds to determine whether the findings hold across demographic variables.

In addition, samples from communities without ready access to the internet would be important to evaluate since internet access and other amenities likely to predispose participants towards digital consumption of information may be lacking, so that students in such communities may be less experienced or less prepared to read texts digitally. This could lead to different patterns of reading preference, experience, and relative advantage; for example, less familiarity with digital media could be associated with less robust semantic memory structures established for information presented in this medium, therefore resulting in lower processing efficiency.

Our participants were middle-school children in the New York City metropolitan area, mostly reporting post-secondary parental education and mid-to-high SES backgrounds. Although the age range of participants was tightly constrained (mean age 10.88 years, SD 0.77), a spread of grade levels (4^th^ to 8^th^) was represented. To minimize the possibility that there could have been differences in reading comprehension due to age or grade, we also confirmed that all participants performed within 3 standard deviations of one another on multiple standard behavioral measures of reading-related skills as quantified by age and grade norms. In addition, stimulus passages used in the experiment were evaluated to ensure accessibility for the ages and grades of our anticipated participants. This text accessibility was confirmed via the analyses of responses to the SVT comprehension measure, confirming no significant interaction between medium of presentation and accuracy across question types. Only the main effect of question type was significant, suggesting that all the passages were easily accessible to our target participants. However, for future study samples we would aim to minimize the spread across grades, as well as the spread of ages.

Our entire sample was born after 2010, and so all can be considered “digital natives” or members of “iGen” (in the sense defined by Twenge et al. [88]). This strongly suggests that digital exposure would have been optimal for these participants throughout their lives, predisposing them to be expert consumers of text and other kinds of information in digital formats. It is also possible that our sample may have been taught or absorbed strategies for reading and learning online given the prevalence of online schooling in New York City during the pandemic that preceded our data collection. Within the current sample, there were no significant differences between the medium of presentation in comprehension of the texts, reading times, or performance on a measure of information retention. Nonetheless, the N400 effects remain; while our findings suggest differences in the efficiency of neurocognitive processing across different media, further research is needed. Overall, the underlying nature of the interaction between experience with particular media and reading comprehension remains to be addressed.

Despite earlier debates about the context of digital adaptations in learning and differences in access to digital media (summarized in [89]), iGen access and exposure to digital media appears uniform across gender, race/ethnicity, and socioeconomic status [88]–even leading to concerns that there has been a displacement of so-called “legacy media” (a term encompassing everything from print books and magazines to television). Carr [90] and Wolf [91] have also suggested that the seemingly shallow processing associated with accessing texts in digital formats could relate to readers being primed by the larger culture of the digital age, to access information in smaller “bits” and to process it less deeply when reading from a screen. Despite such concerns, the majority of our sample identified a preference for print over digital media (similar to that observed in [12]), and we observed a corresponding print advantage in the N400 data for semantic processing of text-related concepts.

Our study parameters were necessarily delimited in many ways. We selected middle-school children for our cross-sectional study design, to reflect the age at which brain adaptations for successful attainment of reading skills are considered to be underway [92, 93]. Chall [94] identified our selected age range as critical in reading development, having proposed a shift in fourth grade from “learning to read” to “reading to learn”–based on the proposal that early learning of basic reading-related skills (such as grapheme-to-phoneme correspondences) shifts around this age to higher-level skills including reading comprehension. Hence, considerations of earlier stages in reading development, and how these adaptations interact with exposure to texts in different mediums, limit the generalizability of our findings.

Other neurophysiological approaches to understanding reading development provide evidence to suggest that a focus on older age groups could also be relevant for future work. For example, Coch [95] used the N400 to investigate orthographic, semantic, and phonological processing in children from 3rd-5th grade, as well as college-age students. Participants were presented with real words, pseudowords, non-pronounceable letter strings, false font strings, and animal names. While an adult-like response was observed for stimuli tapping into semantic and phonological processing, the child participants (but not the college students) showed responses to false font strings similar to their word reading responses. Coch proposed that this changes by adulthood due to extensive reading experience and fine-tuned word processing; but it is not clear at what age automaticity might be attained and what specific neural processes might index such attainment. Until recently, there has been a paucity of evidence-based support for pedagogical practice and policy (e.g., [96]); hence, there is a need to evaluate the application of neurophysiological measures to support effective approaches to developing skilled deep readers.

Another study limitation is instantiated in the limited number of standardized measures conducted to ensure that participants were typically developing readers for their grade and age. Time constraints related to the anticipated average attention span of our target population prohibited the inclusion of other potentially valuable measures. In the future, measures of vocabulary and reading experience could offer deeper insights regarding individual differences. Additionally, we generated recall and retention comprehension question as one measure to ensure equivalency across passages. Unfortunately, missing data from both the recall and retention assessments, compounded by the fact that there were only two items for each question type, made comparisons with the N400 mean amplitude measure difficult.

Despite our best efforts, there were additional limitations and delimitations affecting the experimental design that could have introduced confounds into the study. For example, the presentation of probe words in our experimental paradigm followed reading of text passages in different media, permitting close control of timing that allows for elicitation of event-related brain responses. This design decision was made to facilitate insights into depth of processing that followed reading in different media, and not to identify signatures of brain activation that might differ during actual reading in different text presentation conditions. The presented evidence indicates that we were successful in that goal, but future analysis of EEG, rather than ERP, data recorded during passage reading may yield additional insights. In addition, the text presentation part of the task varied between media (print vs. digital), while the probe word classification task was always presented on a screen. This introduced a difference in congruity between conditions, whereby participants switched from page to screen in the print condition, but did not make a corresponding switch in the digital condition. Since the N400 is understood to index aspects of processing difficulty [52], this difference in congruity between conditions might have introduced a source of systematic variance. Our findings indicate no significant differences between the text presentation media when we evaluated responses to the related and unrelated words, however; the only significant difference in responses between conditions was to the processing of chimera words. Hence, it seems unlikely that this particular potential confound affected our data.

Selection of the Sentence Verification Task as a means to evaluate comprehension may also represent a study delimitation. Performance on the SVT, used as a measure of passage comprehension, was relatively low in both text conditions. The written passages and the SVT test items were controlled on multiple readability measures, so we cannot attribute low scores on the task to the readability or age appropriateness of the texts or of the comprehension measure itself. However, there are other factors that likely contributed. Standard SVT scoring is based on four items per testing category, whereas our version had only two items per category. Nine datasets were missing from the comprehension responses due to equipment failure issues, which could have affected the analyses. The novelty of the SVT method, especially with the full-sentence response anchors we applied, may have rendered the task itself too difficult for our participants, who may have been more accustomed to True/False, Multiple Choice, or Cloze test formats. We continue to consider whether the SVT is a viable means to assess reading comprehension for this target population in a way that is aligned with the depth of processing hypothesis [38]. However, we do note that the SVT responses yielded patterns that could reflect the relatively shallow processing of all the texts in this study–even though differences in *relative* depth of processing were supported by the medium of text presentation and indexed by the children’s brain responses. Specifically, participants scored above chance on both SVT items (explicit and unrelated) that were developed to correlate with shallower processing [42]. Performance on the explicit items did not rise to the 75–80% threshold that, for the standard (non-adapted) version of the SVT, would suggest average to good comprehension performance [61]; but performance on the unrelated SVT items did meet that threshold. For the two test item types developed to correlate with deeper processing, the paraphrase and meaning change items, performance was not as strong (responses to paraphrase items, the easier of the two, were slightly above chance whereas responses to the meaning change items fell below chance).

Similarly to SVT performance, accuracy of probe word classification was quite poor, especially of the Related words. However, accuracy is only one of the study indices of probe word classification; reaction time data and ERP findings offer additional perspectives. Probe word classifications were based on semantic classifications carried out by adults, not children, and the task for semantic classification differed between the adult raters and the child participants. Zielinski et al. [97] used network-level structural covariance analysis to demonstrate that semantic networks related to language continue to develop during childhood; in early adolescence, long-range covariance increases and continues to do so into the teenage years. Therefore, we would expect knowledge of the relationships between words (or knowledge of words) represented within neural networks to vary within our target population and to differ from that of adults, rendering “accuracy” a spurious measure of classification.

Reaction time data, however, did afford some insights into the ways that the child participants processed the probe words. Most telling is the patterning of reaction times between chimera and related words in the print condition, and between chimera and unrelated words in the digital condition; an expected finding following our study predictions, and a precursor to the patterns observed in the ERP analyses. Finally, the relatedness of probe words to preceding passages was captured most effectively by the N400 responses elicited from our participants. The N400 responses to the chimera condition tracked with responses to related words following text reading in the print medium, and with responses to unrelated words following text reading in the digital medium.

Such differences between behavioral (accuracy, reaction time) and neurophysiological (ERPs) indices of behavior are not unexpected in this context. ERPs can reveal mental processes that are difficult to assess behaviorally in children and other populations, and have demonstrated utility as indices of cognitive, affective, or perceptual processes that may not be evident from overt behaviors (e.g., see [98]). Such mismatches between observable behaviors and indices of related brain responses have also been observed in reading studies. For example, Kretzschmar et al. [12] showed that stated preferences of participants for digital media when reading did not negate the ERP and eye-tracking evidence for a print advantage. In the current study, the finding of low accuracy in the semantic classification task across all mediums of presentation likewise does not negate the ERP evidence for deeper lexico-semantic processing of words following text presentations on paper.

During our development of the text passages used as stimuli in this study, we made a decision to work with expository or informational texts. This decision was based on meta-analyses [1, 2] showing that reading performance advantages when reading printed text on paper versus digital formats held more consistently for expository and informational texts than for narrative texts. The selection of expository text allowed us to more effectively control propositional counts for each passage, and to develop passages similar to those likely encountered by children in their learning environments. However, it is possible that distinct effects on indices of neural engagement, and/or behavioral indices of comprehension, could be identified if the texts were narrative in nature. Comparisons between responses to matched sets of narrative and expository texts would be valuable in future work.

## Conclusions

As we have described here, in this study we systematically applied neurophysiological methods to understand the implications and neural underpinnings of reading in print vs. digital media, at a crucial stage in literacy acquisition. An important question raised by these findings concerns the implications for classroom instruction of reading and learning via paper-based texts compared to texts delivered on digital platforms. The question is particularly relevant given the near ubiquitous use of digital platforms for delivery of instruction and information at school and at home.

For reasons related to study delimitations and limitations we think it too early to generate a set of recommendations for adaptation in the classroom. However, we do think that these study outcomes warrant adding our voices to those of Delgado et al. [2] in suggesting that we should not yet throw away printed books, since we were able to observe in our participant sample an advantage for depth of lexical-semantic processing when reading from print. Applications for digital reading should not be dismissed, either: the observation of a potential print advantage does not negate the value of rapid access to information that could be supported by digital reading. It may be that classroom practices should strategically match reading strategies and mediums to task, such that printed media are employed when deeper processing is required while digital access to text is utilized for other needs.

Another reason not to dismiss digital reading platforms is their potential to benefit children with reading disabilities. Research in this area suggests that digital reading strategies may be utilized in support of reading proficiency [99] and comprehension [100] in this population. However, reading disabilities are vastly heterogeneous, and there are concomitant difficulties with identification (e.g., [101]), alongside a corresponding array of interacting causal mechanisms that need to be described at multiple levels—at least, behaviorally, neurophysiologically, and genetically (e.g., [102]). Hence, further investigations of the effectiveness of digital and print text presentations for dyslexia and other reading disabilities will be needed.

Finally, in this study, we predicated a relationship between the activation of semantic networks related to text propositions, and a subsequent classification of those relationships instantiated in single word presentations. We observed differences in brain responses indexing the relatedness of single words to previously-presented texts in print and digital mediums. These data were suggestive of lexical-semantic information being processed in the context of a previously-established network of related concepts, and may indicate that the extent to which such processing is facilitated depends, among other variables, on the medium of text presentation. However, in general, much more work is needed to elucidate the relationships between concepts, words, and the comprehension of discourse or texts.

## Supporting information

S1 File Details of the additional GLMM analysis confirming no effects of participant and item variance on fixed factors medium and word category. (DOCX)

S2 File Details of the correlational analyses between performance on the each of the working memory assessments (Digit Span Forward, Digit Span Backward, and the LSST) and accuracy on the SVT items, both immediate and delayed. (DOCX)
