# Supplementary material for: Middle-schoolers’ reading and lexical-semantic processing depth in response to digital and print media: An N400 study
Source: PLoS One. 2024 May 22;19(5):e0290807. doi: 10.1371/journal.pone.0290807 (PMC11111009; doi:10.1371/journal.pone.0290807)
Supplement: S1 File — (DOCX) [file pone.0290807.s001.docx]

**(G)LMM Supplemental Analysis for Experimental Behavioral Findings**

Although the assumptions and conditions for a two-way repeated-measures ANOVA were met, we conducted additional generalized linear mixed model analyses to confirm that participant and item variance did not reduce the effects of any fixed factors. To structure the models, we did not include effects of age or reading comprehension as covariates, because these were separately controlled by restricting the age of our sample and by excluding participants who were three standard deviations or more away from average scores on standardized reading and language measures. Additionally, we did not expect effects of medium or word probe category to vary from participant to participant. Thus, we constructed linear mixed models for reaction time, probe word accuracy, and SVT item accuracy; with medium, category, and their interaction as fixed effects; and participant and item as random intercepts. In the case of probe word and SVT item accuracy, generalized linear mixed models were applied with a logit link function. Due to the exploratory nature of the delayed data as well as the decreased sample size, such analyses were not performed for those data.

For the linear mixed model with reaction time as the outcome variable, the *mixed* function from the *afex* package in R [1] was used to conduct systematic likelihood ratio tests of the fixed factors and their interaction. These tests did not detect a significant main effect of medium (χ^2^ (1) = 0, *p* = .977); however, they did reveal a significant main effect of category (χ^2^ (2) = 45.02, *p* < .001) and a significant interaction (χ^2^ (2) = 8.47, *p* = .014). These results are concordant with the repeated-measures ANOVA reported in our main document. Additionally, estimates for coefficients within the two models were similar in magnitude (see Table 1 below). The random effects of participant and item, therefore, did not exert any influence on reaction time.

**Table 1. *Reaction Time:* Average RTs Compared Across ANOVA and Mixed Effects Models.**

| **Predictor(s)** | **Average RT, ANOVA** | **Average RT, Mixed Effects Model** |
| --- | --- | --- |
| Digital Related | 1547.06 | 1546.34 |
| Digital Chimera | 1454.83 | 1454.24 |
| Digital Unrelated | 1352.92 | 1353.62 |
| Print Related | 1502.55 | 1502.32 |
| Print Chimera | 1530.48 | 1531.89 |
| Print Unrelated | 1319.92 | 1318.36 |

For probe word accuracy, results again mirrored those of the ANOVA reported in our main document. Likelihood ratio tests indicated only a significant main effect of category (χ^2^ (2) = 768.92, *p* < .001); the main effect of medium was not significant (χ^2^ (1) = .17, *p* = .681) nor was the interaction (χ^2^ (2) = .42, *p* = .809). Similar results were obtained for SVT item accuracy. Likelihood ratio tests indicated only a significant main effect of category (χ^2^ (3) = 36.87, *p* < .001); the main effect of medium was not significant (χ^2^ (1) = .67, *p* = .413) nor was the interaction effect (χ^2^ (3) = 4.91, *p* = .179). This overall relationship between the factors is the same as for the ANOVA conducted. Parameter estimates for all three mixed models can be found in the following tables (2–4).

**Table 2. *Reaction Time:* Parameter Estimates**

| **Model Terms** | **Parameter Estimate β (SE)** |
| --- | --- |
| Intercept (Digital Chimera) | 1454.24 (61.38) |
| Print | 77.65 (33.02) |
| Related | 92.10 (37.27) |
| Unrelated | -100.62 (37.22) |
| Print * Related | -121.67 (46.63) |
| Print * Unrelated | -112.89 (46.62) |

**Table 3. *Probe Word Accuracy:* Parameter Estimates**

| **Model Terms** | **Parameter Estimate β (SE)** |
| --- | --- |
| Intercept (Digital Chimera) | -2.13 (0.15) |
| Print | 0.004 (0.08) |
| Related | 1.91 (0.13) |
| Unrelated | 6.05 (0.17) |
| Print * Related | -0.06 (0.10) |
| Print * Unrelated | -0.02 (0.16) |

**Table 4. *SVT Item Accuracy:* Parameter Estimates**

| **Model Terms** | **Parameter Estimate β (SE)** |
| --- | --- |
| Intercept (Digital Explicit) | 0.69 (0.32) |
| Print | 0.05 (0.18) |
| Meaning Change | -1.20 (0.46) |
| Paraphrase | -0.68 (0.45) |
| Unrelated | 0.85 (0.46) |
| Print * Meaning Change | -0.30 (0.28) |
| Print * Paraphrase | 0.04 (0.26) |
| Print* Unrelated | 0.38 (0.29) |

**Reference**

1. Singmann H, Bolker B, Westfall J, Aust F, Ben-Shachar M-S, Højsgaard S, Fox J, Lawrence MA, Mertens U, Love J, Lenth R, Christensen RHB. (2024). *afex: Analysis of Factorial Experiments. R package version 0.16-1.* https://CRAN.R-project.org/package=afex
