# Supplementary material for: Middle-schoolers’ reading and lexical-semantic processing depth in response to digital and print media: An N400 study
Source: PLoS One. 2024 May 22;19(5):e0290807. doi: 10.1371/journal.pone.0290807 (PMC11111009; doi:10.1371/journal.pone.0290807)
Supplement: S2 File — (DOCX) [file pone.0290807.s002.docx]

As reported in the main text, to determine whether performance on the SVT was affected by working memory, we calculated correlations between performance on the each of the working memory assessments (Digit Span Forward, Digit Span Backward, and the Listening Sentence Span Task – LSST [1]) and accuracy on the sentence verification task (SVT) items, both immediate and delayed. Spearman correlations (Bonferroni-corrected for multiple comparisons) were used due to the ordinal nature of the LSST and possible non-normal distributions of the variables. The only correlations that remained significant when applying the Bonferroni correction were between LSST scores and immediate SVT scores, both total (*r* = .459, *p* = .000801, corrected *p* = .036) and responses to the paraphrase items (*r* = .467, *p* = .0006287, corrected *p* = .028).

Table 1 below provides *r*-statistics, uncorrected *p*-values, and Boferroni-corrected *p*-values for correlations between immediate SVT scores and three assessments of working memory. Table 2 below provides the same information for the delayed SVT scores.

**Table 1. Spearman Correlations and *p*-Values for Immediate Scores on the Sentence Verification Task and Working Memory Assessments.**

|  | *Sentence Verification*  *Item Type* | *Digit Span Forward*  *(p uncorrected; p corrected)* | *Digit Span Backward*  *(p uncorrected; p corrected)* | *Listening Sentence Span Task*  *(p uncorrected; p corrected)* |
| --- | --- | --- | --- | --- |
| *ALL ITEMS* | *Explicit* | .031  (.833; 1.00) | -.226  (.114; 1.00) | .071  (.623; 1.00) |
|  | *Paraphrase* | .108  (.457; 1.00) | .125  (.386; 1.00) | .467  (.0006*; .028*) |
|  | *Meaning Change* | .011  (.938; 1.00) | .212  (.140; 1.00) | .076  (.601; 1.00) |
|  | *Unrelated* | .176  (.221; 1.00) | .172  (.232; 1.00) | .245  (.087; 1.00) |
|  | *All Types* | .128  (.377; 1.00) | .128  (.377; 1.00) | .459  (.0008*; .036*) |
| *DIGITAL* | *Explicit* | -.073  (.614; 1.00) | -.319  (.024*; 1.00) | .128  (.375; 1.00) |
|  | *Paraphrase* | .088  (.543; 1.00) | .233  (.104; 1.00) | .413  (.003*; 1.00) |
|  | *Meaning Change* | -.069  (.636; 1.00) | .019  (.895; 1.00) | -.151  (.295; 1.00) |
|  | *Unrelated* | .057  (.695; 1.00) | .077  (.596; 1.00) | .216  (.133; 1.00) |
|  | *All Digital* | .032  (.827; 1.00) | .029  (.840; 1.00) | .366  (.009*; 1.00) |
| *PRINT* | *Explicit* | .100  (.491; 1.00) | .028  (.846; 1.00) | -.032  (.826; 1.00) |
|  | *Paraphrase* | .068  (.638; 1.00) | -.014  (.926; 1.00) | .308  (.030*; 1.00) |
|  | *Meaning Change* | .022  (.881; 1.00) | .220  (.124; 1.00) | .177  (.219; 1.00) |
|  | *Unrelated* | .331  (.019; 1.00) | .279  (.049; 1.00) | .247  (.083; 1.00) |
|  | *All Print* | .240  (.093; 1.00) | .198  (.167; 1.00) | .348  (.013*; 1.00) |

**p* < .05; second *p*-value in each cell is Bonferroni-corrected for multiple comparisons.

**Table 2. Spearman Correlations and *p*-Values for Delayed Scores on the Sentence Verification Task and Working Memory Assessments.**

|  | *Sentence Verification*  *Item Type* | *Digit Span Forward*  *(p uncorrected; p corrected)* | *Digit Span Backward*  *(p uncorrected; p corrected)* | *Listening Sentence Span Task*  *(p uncorrected; p corrected)* |
| --- | --- | --- | --- | --- |
| *ALL ITEMS* | *Explicit* | .219  (.112; 1.00) | .161  (.246; 1.00) | .044  (.752; 1.00) |
|  | *Paraphrase* | .094  (.498; 1.00) | .078  (.564; 1.00) | .187  (.175; 1.00) |
|  | *Meaning Change* | .160  (.249; 1.00) | .104  (.456; 1.00) | .154  (.267; 1.00) |
|  | *Unrelated* | .038  (.785; 1.00) | -.018  (.899; 1.00) | .191  (.167; 1.00) |
|  | *All Types* | .208  (.131; 1.00) | .106  (.447; 1.00) | .208  (.132; 1.00) |
| *DIGITAL* | *Explicit* | .138  (.321; 1.00) | .095  (.494; 1.00) | -.176  (.204; 1.00) |
|  | *Paraphrase* | .052  (.710; 1.00) | -.051  (.713; 1.00) | .048  (.733; 1.00) |
|  | *Meaning Change* | .063  (.653; 1.00) | -.051  (.716; 1.00) | .075  (.592; 1.00) |
|  | *Unrelated* | -.022  (.872; 1.00) | -.042  (.761; 1.00) | .164  (.235; 1.00) |
|  | *All Digital* | .080  (.563; 1.00) | -.059  (.672; 1.00) | .011  (.939; 1.00) |
| *PRINT* | *Explicit* | .224  (.104; 1.00) | .166  (.229; 1.00) | .176  (.202; 1.00) |
|  | *Paraphrase* | .118  (.394; 1.00) | .175  (.206; 1.00) | .231  (.092; 1.00) |
|  | *Meaning Change* | .135  (.332; 1.00) | .197  (.153; 1.00) | .127  (.360; 1.00) |
|  | *Unrelated* | .095  (.493; 1.00) | .020  (.884; 1.00) | .221  (.108; 1.00) |
|  | *All Print* | .198  (.152; 1.00) | .205  (.137; 1.00) | .353  (.009*; 1.00) |

**p* < .05; second *p*-value in each cell is Bonferroni-corrected for multiple comparisons.

**Reference**

1. Swanson HL. Generality and modifiability of working memory among skilled and less skilled readers. J Educ Psychol. 1992;84(4):473–88. doi: 10.1037/0022-0663.84.4.473
